# Candle flame soot sizing by planar time-resolved laser-induced incandescence

**DOI:** 10.1038/s41598-020-68256-z

**Published:** 2020-07-09

**Authors:** Ignacio Verdugo, Juan José Cruz, Emilio Álvarez, Pedro Reszka, Luís Fernando Figueira da Silva, Andrés Fuentes

**Affiliations:** 1 Departamento de Industrias, Universidad Técnica Federico Santa María, Av. España 1680, Casilla 110-V, Valparaiso, Chile; 2 Faculty of Engineering and Sciences, Universidad Adolfo Ibáñez, Santiago, Chile; 3 Department of Mechanical Engineering, Pontifícia Universidade Católica do Rio de Janeiro, Rua Marquês de São Vicente, 225, Rio de Janeiro, RJ 22.451-900 Brazil

**Keywords:** Environmental sciences, Energy science and technology, Engineering, Nanoscience and technology, Optics and photonics, Physics

## Abstract

Soot emissions from flaming combustion are relevant as a significant source of atmospheric pollution and as a source of nanomaterials. Candles are interesting targets for soot characterization studies since they burn complex fuels with a large number of carbon atoms, and yield stable and repeatable flames. We characterized the soot particle size distributions in a candle flame using the planar two-color time-resolved laser induced incandescence (2D-2C TiRe-LII) technique, which has been successfully applied to different combustion applications, but never before on a candle flame. Soot particles are heated with a planar laser sheet to temperatures above the normal flame temperatures. The incandescent soot particles emit thermal radiation, which decays over time when the particles cool down to the flame temperature. By analyzing the temporal decay of the incandescence signal, soot particle size distributions within the flame are obtained. Our results are consistent with previous works, and show that the outer edges of the flame are characterized by larger particles ($\approx 60\,\hbox {nm}$), whereas smaller particles ($\approx 25\,\hbox {nm}$) are found in the central regions. We also show that our effective temperature estimates have a maximum error of 100 K at early times, which decreases as the particles cool.

## Introduction

Candles are one of the oldest combustion technologies still in use. They represented a significant technological advancement over oil lamps, including the lack of dripping and the ability to produce a stable flame due to its self-trimming wick. Ever since Michael Faraday’s famous lectures^1^, and albeit their deceptive simplicity, candle flames remain perhaps the most archetypal non-premixed combustion system, and have received continuous attention from the combustion community^2–7^. As practical combustion systems, candles include several complex processes in the solid, liquid and gaseous phases in a compact and safe setting. The paraffin wax is held in the system as a solid and is only liquefied before it is fed to the flame zone, which represents an important attribute in terms of safety, storage and transportation. Liquid fuel is fed to the flame through the wick by way of capillary movement. As the fuel reaches the top of the wick, it evaporates and the gaseous fuel diffuses towards the reaction zone, a thin surface located at the exterior of the flame where the combustion reactions take place, while it undergoes thermal decomposition. In terrestrial gravity conditions, the buoyant, hot gases generated by the energy released in the combustion reactions move upward, entraining fresh air into the reaction zone, ensuring the sustained combustion in the system. Gravity thus gives the flame its characteristic shape, and candles have been the subject of significant research in microgravity conditions by NASA^3,8^. Perhaps the main feature of candle flames is their luminosity, and nowadays they are still an important light source, particularly for the approximately 800 million people still living without access to electricity^9^. Candle flame luminosity is due to the emission of thermal radiation by incandescent carbon-based nanoparticles, commonly known as soot. These particles are produced within the reaction zone by a complicated set of chemical reactions^10,11^ and represent an important source of particulate matter pollution. Considering a global candle market worth several billion US dollars, it can be concluded that candles are a ubiquitous source of domestic pollution. Health effects are now understood to be dependent on the maturity of the emitted soot particles^12,13^, i.e., the stage in the soot formation process during which the particle leaves the reaction zone. The morphology and chemical structure of the particles depend on their maturity and have an important effect on the way these particles interact with the tissues in the respiratory tract. There is solid scientific evidence indicating that exposure to particulate matter has harmful cardiopulmonary effects^13^. Detailed soot characterization becomes an important element when assessing the health effects of combustion systems, ranging from domestic devices like candles, heaters and cookers, to industrial equipment such as biomass boilers. Soot characterization also contributes to address the environmental concerns of particulate matter emitted by practical combustion systems^14^, which should comply with an ever stringent set of environmental requirements, including reduced particulate emissions. In order to achieve this, fuel chemistry and soot formation numerical models must be validated with experimental data, with a focus on sooting propensity and soot morphology^15–17^. Candle flames are laminar and generally stable, offering the possibility of carrying out non-intrusive, laser-based diagnostics to study sooting propensity and morphology on an axisymmetric geometry. Therefore, these flames become an interesting target for performing soot characterization measurements, since they yield stable and repeatable flames^6^. A previous study shows that the chemical composition of soot at flame tip consists of 89 atom % C and 11 atom % O (mainly ultrafine particles of elemental carbon and ash), whereas the inner flame consists of 91 atom % C and 9 atom % O (large particles and aggregates)^18^. The goal of this article is thus to present insight on soot production processes within candle flames while introducing a state-of-the-art soot characterization technique to a multi-disciplinary audience with an interest in the broader implications of particulate matter emissions.

Previous work has shown the capabilities of laser-based diagnostic techniques to shed new light on the soot production processes within candle flames. Specifically, soot concentrations and temperatures have been measured in candle flames, showing that the wick diameter controls the soot volume fraction^6^. Although soot morphology from candle flames has been studied using intrusive^18,19^ and local non-intrusive techniques^20^, no studies have yet reported field measurements of soot morphology. This paper is thus devoted to the experimental characterization of soot particle diameter and temperature in controlled burning candle flames. The particle diameter measurement is effected by using time-resolved laser induced incandescence (TiRe-LII), which is a well-established and accepted non-intrusive technique available for this purpose^21^.

Laser induced incandescence (LII) is an in-situ non-intrusive diagnostic which allows studying soot formation^22^, as well as soot concentration measurements^23^. In this technique, the incandescence of the soot particles is attained by heating the particles up to $\approx 4,000$ K with laser irradiation. If the incandescence signal is temporally analyzed, soot particle size distributions can be obtained. This technique, known as Time Resolved LII (TiRe-LII), has been successfully applied in several experimental configurations^24–27^. Since different sized particles will have different thermal inertia, the incandescence signal ($\text {S}_{\text {LII}}$) from smaller particles decays more rapidly than the signal from larger particles. Particle size distribution is inferred by employing laser fluences that are lower than those used for for applying the classic LII technique, actually decreasing the soot sublimation effects, and allowing an energy balance equation applicable to this problem to be presented and solutions to be obtained iteratively to account for the different particle sizes. In this case, an effective soot particle aggregate temperature is measured using time-resolved two-color optical pyrometry, which is then compared with a numerical LII model to infer a mean soot particle size^28,29^. Note that the effective temperature represents the instantaneous peak temperature attained by a soot particle ensemble after the laser heating. The effective temperature decays with time after the laser pulse. Typically, TiRe-LII measurements have been carried out with photo-multiplier tubes (PMTs)^20,30^, which provide good temporal resolution over hundreds of nanoseconds, but limits the analysis to point measurements. To overcome this shortcoming, planar TiRe-LII measurements use intensified cameras (ICCD) to capture the incandescence signals at different delay times after the laser pulse^24,31–33^.

## Results

### Primary soot particle temperature

**Fig. 1:**
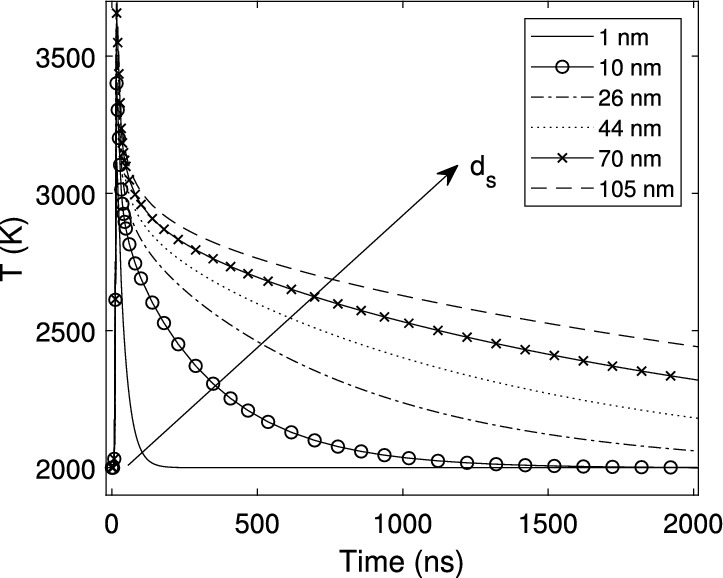
Theoretical temperature decay of different soot primary particle diameters, $\text {d}_\text {s}$.

As a first analysis step, the energy balance equation of a single soot particle (Eq. 1) in the Methods section below) is solved for spherical particle diameters in the range of values between 1 and 105 nm. The assumed initial particle temperature (2,000 K) employed in the iterative processes, is a typical diffusion flame temperature, and the applied fluence during 8 ns is $0.148\,\text {J/cm}^2$. The resulting theoretical temperature histories may be observed in Fig. 1, where the very short initial heating period that occurs during the laser pulse heats the soot particles to temperatures greater than 3,000 K. This figure shows that the temperature decay time of the smallest soot particles is on the order of a few nanoseconds, whereas the largest particles show temperatures exceeding 2,500 K even after $2\,\upmu\hbox {s}$. It is then clear that the temporal temperature decay rate may be used for the purposes of discriminating between primary soot particles with diameters spanning several orders of magnitude, as previously underscored by several works^27,28,32,34^. Note that, in the framework of the TiRe-LII technique, the calculated temperature is used to determine the theoretical effective temperature, $\text {T}_{\text {e}}(\text {d}_{\text {pg}},\sigma _{\text {g}})$, through Eq. (10), where $\text {d}_\text {pg}$ and $\sigma _\text {g}$ are the geometric mean particle diameter and the geometric standard deviation of an assumed lognormal particle size distribution^35^.

### Temporal signal decay

**Fig. 2:**
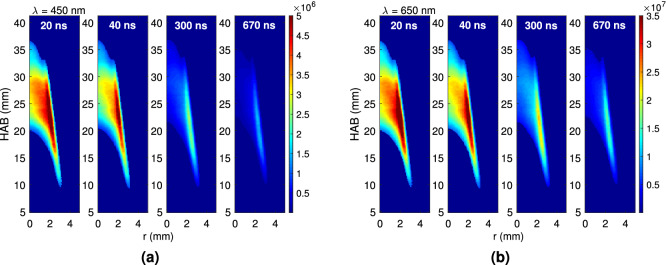
Temporal decay of incandescence signals, measured at 20, 40, 300 and 670 ns after the laser pulse, for the two used detection wavelengths. (**a**) Fields of $\text {S}_{\text {LII}}$ (a.u.) at 450 nm and (**b**) Fields of $\text {S}_{\text {LII}}$ (a.u.) at 650 nm.

The temporal decay behavior of the raw LII signals is the fundamental quantity used in the TiRe-LII technique. Figure 2 presents this decay along the flame for the studied candle. Note that since axial symmetry is assumed, only one half of the candle flame directly exposed to the laser signal is presented here, and that the origin of the vertical coordinate, HAB (Height Above the Base), lies at the candle wax pool surface.

Each of the LII intensity fields given in this figure is the outcome of averaging 200 instantaneous images, each of which corresponds to the the ICCD camera gated at 20 ns. During the experiments the camera opening delay with respect to the laser pulse was varied from 20 ns to 1,000 ns. In Fig. 2 representative images of the decay process are depicted, taken at the delays of 20, 40, 300 and 670 ns. These delay values have been chosen so as to represent the prompt, short time, intermediate time and long term LII signal behaviour. Figure 2a, b also give the temporal decay at the two detection wavelengths used in this study, i.e., $\lambda = 450$ and 650 nm, respectively. These detection wavelengths have been chosen to avoid fluorescence from gas-phase flame species (PAH and others)^36,37^, to account for the camera spectral sensitivity – thus yielding comparable LII signal intensities—and to provide an adequate spectral separation. Indeed, the emission intensity of the particles is higher for $\lambda _2 = 650\,\hbox {nm}$ than for $\lambda _1 = 450\,\hbox {nm}$^38^, compensating for the signal ratio loss due to the spectral quantum efficiency variation of the ICCD camera.

**Fig. 3:**
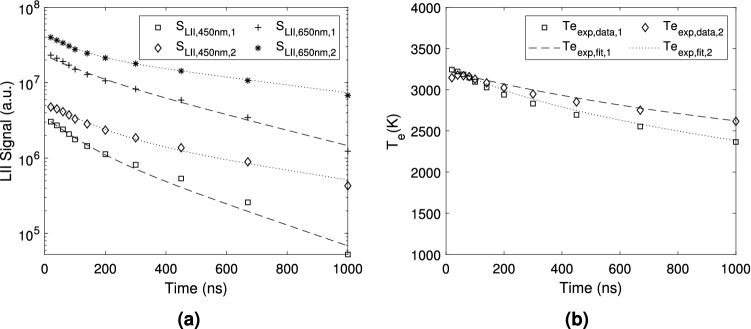
Decay of the LII signals and of the effective temperature at two different regions of the candle flame: (1) Centerline—$\hbox {r} = 0\,\hbox {mm}$ and $\hbox {HAB} = 28\,\hbox {mm}$ and (2) $\text {f}_\text {v,max} - \hbox {r} = 2\,\hbox {mm}$ and $\hbox {HAB} = 22.5\,\hbox {mm}$: (**a**) $\text {S}_{\text {LII}}$ at 450 nm and 650 nm and (**b**) Experimental effective soot temperature ($\text {T}_\text {e,exp}$).

The LII signal is larger for the longer wavelength, with local signal maxima at each HAB, possibly highlighting the position of the higher soot volume fraction ($\text {f}_\text {v}$). The LII intensities are highest at 20 ns, with a peak region located around HAB $\approx 20\,\hbox {mm}$. Therefore, this particular high intensity region is used here for analysis purposes, since it may be assumed that a fixed proportionality factor between the $\text {S}_{\text {LII}}$ intensity and $\text {f}_\text {v}$^17,21^ exists. Figure 3 presents the decay history of the LII signal for the two measurement wavelengths, i.e., $\text {S}_{\text {LII}, 450}$ and $\text {S}_{\text {LII}, 650}$, at two different locations along the flame, as well as the corresponding effective soot temperature. These regions correspond to the position along the flame centerline where the maximum LII signal is observed and to the location of maximum soot volume fraction ($\text {f}_\text {v,max}$). In both regions, $\text {S}_{\text {LII}, 450} < \text {S}_{\text {LII}, 650}$ (cf. Fig. 3a). Furthermore, at each wavelength, as expected, the signal at the maximum soot volume fraction region is larger than that at the chosen centerline position. Figure 3b also depicts an exponential fit of the experimental effective temperature $\text {T}_{\text {e,exp}}$, which is needed for comparison purposes with that numerically calculated $\text {T}_{\text {e}}(\text {d}_{\text {pg}},\sigma _{\text {g}})$, and to obtain the *lognormal* diameter distribution parameters. The experimental effective temperature [cf. Eq. (11) below] is found to decay monotonically from 3,200 to 2,600 K, as may be verified in Fig. 3b. These temperature values, lower than the carbon sublimation point, are consistent with the desired application of the TiRe-LII technique.

### Soot temperature and diameter

**Fig. 4:**
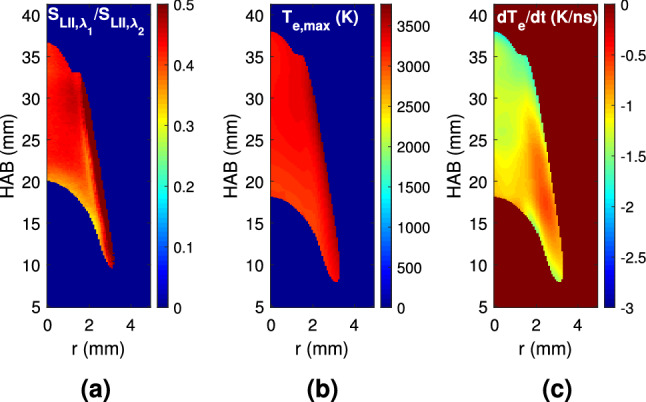
Fields of (**a**) Incandescence signal ratio ($\text {S}_{\text {LII,450}}/\text {S}_{\text {LII,650}}$) at 20 ns, (**b**) maximum effective temperature ($\text {T}_{\text {e,max}}$) and (**c**) fitted temperature decay rate $\text {dT}_{\text {e}}/\text {dt}$, Eq. (12) between 20 and 100 ns.

In order to further characterize the LII signal at early times, Fig. 4 gives the fields of the signal ratio at the two detection wavelengths ($\text {S}_{\text {LII},\lambda _1}/\text {S}_{\text {LII},\lambda _2}$), the maximum effective temperature computed at 20 ns and the fitted temperature decay rate between 20 and 100 ns. The signal ratio varies between 0.3 and 0.5, with the smaller values obtained at lower portion of the flame, closer to the wick, and the larger being characteristic of the outer, oxidizing, region. The effective temperature at this early time is as high as 3,200 K at the outermost regions of the flame, and descends to $\approx$3,000 K towards the flame axis. This rather uniform effective temperature distribution indicates that, regardless of their diameter or concentration, the soot particles are being heated to similar temperatures by the laser pulse. The corresponding temperature decay rate exhibits a higher absolute value at the innermost, central flame regions, and smaller at the outer regions. Furthermore, the effective temperature peak ($\text {T}_{\text {e,max}}$) and the corresponding distribution, obtained by the two-color technique, are quite similar to previously reported values obtained for comparable laser fluences (3,180 K at a fluence of $0.17\,\hbox {J/cm}^2$)^33^.

**Fig. 5:**
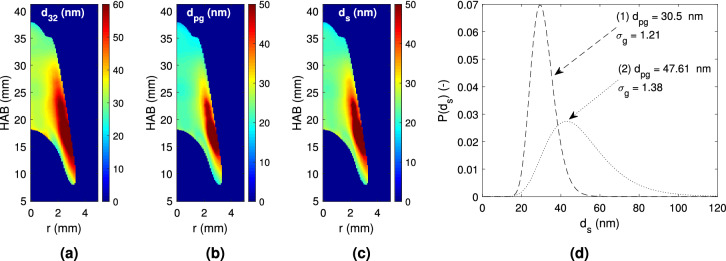
Fields of: (**a**) Sauter mean diameter ($\text {d}_{\text {32}}$), (**b**) Geometric mean diameter ($\text {d}_{\text {pg}}$), (**c**) mean primary soot particle diameter ($\text {d}_{\text {s}}$) and (**d**) primary particle diameter distribution, c.f. Eq. (8). The *Lognormal* particle size distributions show different ($\text {d}_{\text {pg}}$, $\sigma _{\text {g}}$) at two points of the flame: (1) centerline—$\hbox {r} = 0\,\hbox {mm}$ and $\hbox {HAB} = 28\,\hbox {mm}$ and (2) maximum soot volume fraction, $\text {f}_\text {v,max} - \hbox {r} = 2\,\hbox {mm}$ and $\hbox {HAB} = 22.5\,\hbox {mm}$.

The effective temperature decay rate distribution indicates that the larger soot particles should be located in the vicinity of the oxidizing region, and that a rather uniform diameter distribution should arise along the flame centerline. This may indeed be verified in Fig. 5a, b, where the obtained soot particle diameters are given. The Sauter mean diameter ($\hbox {d}_{32}$), is a classical particle size average, defined as the ratio of the third to second moments of the particle diameters distribution^39^, and represents the diameter of a particle whose surface-to-volume ratio is equal to the entire soot particles ensemble at the region of interest^40^. Accordingly, the geometric and Sauter mean diameter values reach 50 and 60 nm at the outermost regions of the sooting region, respectively, whereas smaller corresponding values—35 and 25 nm—arise near $r=0$. In order to gain further insight on the soot particle diameter distribution within the candle flame, Fig. 5c shows the computed values of the primary particle diameter, $\text {d}_{\text {s}}$, obtained by Eq. (8). Attention is drawn first to Fig. 5d, where the probability distributions of primary particle diameter are plotted at two positions within the flame. The first of these positions is characteristic of the flame centerline behavior, and the second of the maximum soot volume fraction location. This figure indicates that a narrower primary particle diameter distribution is observed at the centerline position, i.e., $20< \text {d}_{\text {s}} < 35$ nm, whereas a wider one ($20< \text {d}_{\text {s}} < 90$ nm) characterizes the maximum soot volume fraction region. The corresponding field of primary particle diameter $\text {d}_{\text {s}}$ may be seen in Fig. 5c. These diameters are found to range from 20 nm, near $r=0$, to 50 nm, at the vicinity of the soot oxidation region, where the soot volume fraction is maximum. As expected, the overall diameter spatial distribution is similar, regardless of which – $\text {d}_{\text {32}}$, $\text {d}_{\text {s}}$ or $\text {d}_{\text {pg}}$—is considered (cf. Fig. 5). The primary particles diameter, $\hbox {d}_s$, obtained here are comparable with previous results obtained by TEM images (20–50 nm)^18^ and point TiRe-LII measures ($\approx 55\,\hbox {nm}$)^20^.

## Discussion

In this work, the planar two-color time-resolved laser induced incandescence (2D-2C TiRe-LII) technique was used to characterize a candle flame operating below the smoke point. To the best of the authors’ knowledge, at present there are no works that report soot particle sizes for a candle flame using this technique for two dimensions. Nevertheless, TiRe-LII has been applied to Candle point measurements^20^, *n*-heptane^34^, ethylene^31–33,41^ and ethane^24^ flames, yielding similar results both for the $\text {S}_{\text {LII}}$ and particle diameter distributions. The effective soot temperatures decay from 3,200 to 2,600 K, which is consistent with TiRE-LII applications at the laser fluences used in this study. The larger soot particles, with $\text {d}_{\text {32}}$ $\approx 60$ nm, tend to be located at the outer edges of the sooting region, whereas the flame centerline is characterized by the presence of smaller particles ($\text {d}_{\text {32}}\approx 25\,\hbox {nm}$).

**Fig. 6:**
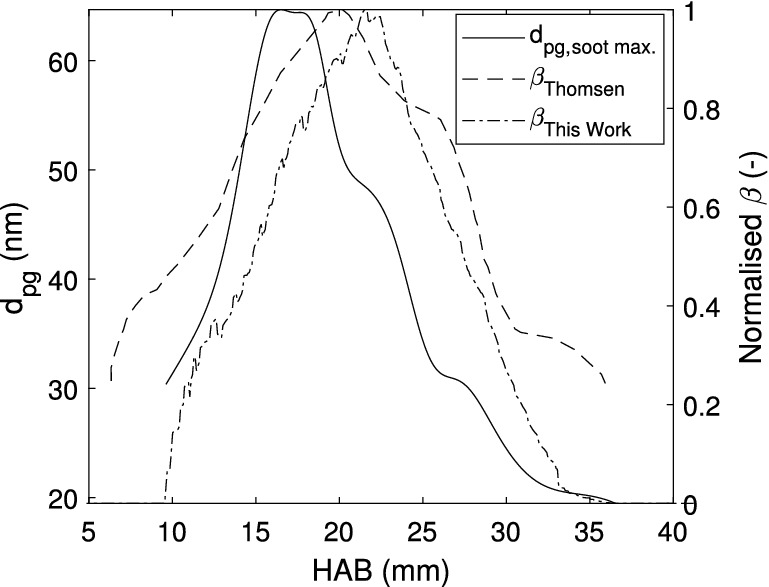
Vertical evolution of the geometric mean diameter ($\text {d}_{\text {pg}}$) along the maximum soot volume fraction (the incandescence field at 20 ns), and the normalized integrated soot volume fraction ($\beta$), compared with previously obtained results^6^.

**Fig. 7:**
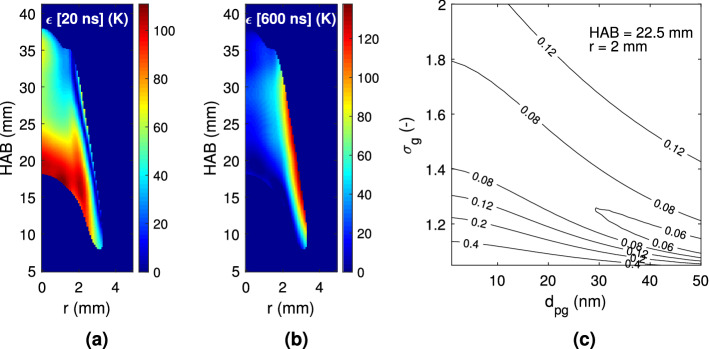
Error analysis results: (**a**) Effective temperature difference $\epsilon$ (Eq. 14) at 20 ns, (**b**) Effective temperature differences $\epsilon$ (Eq. 14) at 600 ns and (**c**) Likelihood estimator ($\chi ^2$, Eq. (15)) at position $\hbox {r} = 2\,\hbox {mm}$ and HAB 22.5 mm.

Figure 6 depicts the vertical evolution of a set of properties characterizing the sooting flame. Following the line of maximum soot volume fraction, this figure presents the geometric mean diameter, which exhibits a non monotonic behavior. Soot particles are first detected with $\text {d}_{\text {pg}} \approx 32\,\hbox {nm}$, at HAB $\approx 9\,\hbox {mm}$, and the diameter is found to increase up to 64 nm at HAB $\approx 17\,\hbox {mm}$. A diameter decrease is then observed until HAB $\approx 35\,\hbox {mm}$, where the detection limit is again reached. Following previous works^6^, this figure also shows the radially integrated soot volume fraction evolution with HAB, normalized with respect to the peak value $\left( \beta = 2\pi \int _{0}^{\infty } \text {f}_\text {v}\text {(r) rdr}.\right)$. Note that from the relationship between $\text {f}_\text {v}$ and $\text {S}_{\text {LII}}$, the normalized $\beta$ values are related to the incandescence signal, which is taken at the prompt detection time, where the signal reaches its maximum value. For this purpose, either $\lambda _1$ or $\lambda _2$ may be used, since $\text {f}_\text {v}$ is a physical property of the flame. The results of the evolution with height of the integrated soot volume fraction are compared in Fig. 6 with previously measured values using Modulated Absorption Emission (MAE) for the same candle flame^6^. The previous measurements detected soot in significant amounts ($\beta = 0.3$) at HAB $\approx 7\,\hbox {mm}$, whereas in this work these amounts are observed further downstream ($\hbox {HAB} \approx 11\,\hbox {mm}$). This discrepancy is related to the different methods used to determine $\beta$, since in the present work $\text {S}_{\text {LII}}$ nearly vanishes below $\hbox {HAB} \approx 11\,\hbox {mm}$, whereas the MAE signal is strong in the lower parts of the flame^6^. The maximum value of $\beta$ is found to occur nearly at the same position ($\hbox {HAB} \approx 20\,\hbox {mm}$) for the two measurements, and the progressive integral soot volume fraction decrease rate with HAB is nearly identical. A final characterization of the TiRE-LII application to the studied candle flame is the temperature and diameter error analysis. To that end, Fig. 7 presents both the effective temperature difference ($\epsilon$), given by Eq. (14), and the normalized likelihood estimator ($\chi ^2$), as defined by Eq. (15). The first figure-of-merit, $\epsilon$, is examined at two different times, 20 ns and 600 ns in Fig. 7a, b, respectively. These figures show that both the magnitude and the distribution of this error change with time. Indeed, at earlier times the maximum error, which is on the order of 100 K, is found to occur at the innermost parts of the measurement region, whereas at later times the maximum is displaced towards the outer edges of this region. Furthermore, at 600 ns, $\epsilon$ is smaller than 50 K at the central part of the flame but, at 20 ns it remains larger than 60 K at the upper parts of the flame centerline. These differences between the experimental and theoretical effective temperatures are mainly associated to the $\text {S}_{\text {LII}}$ measurements. By comparing the signal ratio $\text {S}_{\text {LII},\lambda _1}/\text {S}_{\text {LII},\lambda _2}$, given in Fig. 4a, and $\epsilon$ at 20 ns (see Fig. 7a), it is possible to verify that the largest temperature difference occurs at the area of the smallest signal ratio value, and vice-versa. This underscores the close relationship that exists between the signals ratio and the effective temperature difference. Furthermore, considering the values of $\epsilon$ at 600 ns (see Fig. 7b), it can be deduced from Fig. 3a that the $\text {S}_{\text {LII}}$ ratio decreases with time, so that the difference between the effective temperature is smaller at the selected points. As a consequence, at later times the temperature difference $\epsilon$ is larger at the outer flame zones, where the temperature decays faster, than at the inner regions. The $\chi ^2$ likelihood estimator field depicted in Fig. 7c is evaluated at a radial location of 2 mm from the flame axis and a height of 22.5 mm as a function of the diameter distribution parameters, $\text {d}_{\text {pg}}$ and $\sigma _{\text {g}}$. This position has been chosen because it corresponds to the maximum soot volume fraction region. This figure shows the minimum value of $\chi ^2$ that characterizes the distribution shown in Fig. 5d. The $\chi ^2$ determination has been performed for each pair of parameters, for time intervals ranging from 20 ns to 1 $\upmu$m. The estimate range for $\text {d}_{\text {pg}}$ spans from 0 to 50 nm with a 0.05 nm step, and the corresponding one for $\sigma _{\text {g}}$ is from 1 to 2 with a 0.01 step. Figure 7c indicates that a $\chi ^2$ mathematical minimum exists for the chosen parameter variation of the *lognormal* distribution. In particular, the region of $\text {d}_{\text {pg}}$ from 30 to 50 nm and $\sigma _{\text {g}}$ from 1.1 to 1.3 is where an absolute minimum seems to lie, i.e., where the difference between the values of effective temperatures is the smallest. The parameters of the *lognormal* distribution at this region (see Fig. 5d) are those that minimize the $\chi ^2$-value. However, since a minimum value may not be sharply distinguished, the values obtained above ($\text {d}_{\text {pg}}$ and $\sigma _{\text {g}}$ given in Fig. 5d) are assumed to be representative of those that minimize $\chi ^2$-value.

**Fig. 8:**
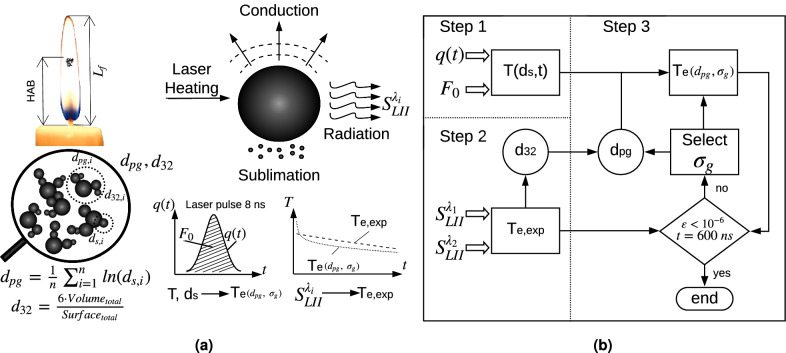
Schematics of the TiRe-LII methodology: (**a**) Heating process and relevant properties: HAB is the height above the base, i.e., measured from the wax pool surface; soot particle and aggregates characteristic dimensions; laser energy temporal evolution; experimental and theoretical effective temperature decays; and (**b**) Flowchart for geometric mean soot particle diameter; Step 1: theoretical temperature decay; Step 2: experimental effective temperature decay and Sauter mean diameter; Step 3: error minimization, lognormal distribution properties.

## Methods

### Theoretical background: the TiRe-LII model

The laser-induced incandescence technique (LII) is widely used to study the formation of soot particles within flames^42^. This technique is implemented by applying a nanosecond laser pulse that increases the soot particles temperature to levels where detectable incandescence signals arise ($\text {S}_{\text {LII}}$)^43,44^. After the laser irradiation, the $\text {S}_{\text {LII}}$ decays as the soot particle temperature returns to the surrounding flame condition. Since smaller soot particles cool down faster than larger ones^22,45^, due to the larger surface area to volume ratio and smaller thermal inertia, the soot particle size distribution may be determined from the temporal decay study of $\text {S}_{\text {LII}}$. This may be performed by comparing the measured soot temperature decay with the computed temperature results obtained by assuming a soot primary particle size probability distribution^21,44^. Please refer to Fig. 8 for a visual summary of the technique. The temperature time-history, $\text {T(t)}$, of a single soot primary particle, with diameter $\text {d}_{\text {s}}$, density $\rho _\text {s}$ (1,900 kg/m$^3$) and specific heat $\text {c}_\text {s}$ with a temperature dependence according to Liu’s model^29^, may be modeled by the energy balance equation^28,29^:

1 $$\frac{1}{6}\pi {\text{d}}_{{\text{s}}}^{3} \rho _{{\text{s}}} {\text{c}}_{{\text{s}}} \frac{{{\text{dT}}}}{{{\text{dt}}}} = {\text{C}}_{{\text{a}}} {\text{F}}_{{\text{0}}} {\text{q}}({\text{t}}) - {{\dot{\text{q}}}}_{{\text{r}}} - {{\dot{\text{q}}}}_{{\text{c}}} - {{\dot{\text{q}}}}_{{\text{s}}} ,$$

which states that the internal energy rate of change is equal to the sum of laser energy absorption, ${\text{C}}_{{\text{a}}} {\text{F}}_{{\text{0}}} {\text{q}}({\text{t}})$, and the energy loss by thermal radiation, ${{\dot{\text{q}}}}_{{\text{r}}}$, conduction, ${{\dot{\text{q}}}}_{{\text{c}}}$, and sublimation, ${{\dot{\text{q}}}}_{{\text{s}}}$. In the laser irradiation term, ${\text{C}}_{{\text{a}}}$ is the absorption cross section, ${\text{F}}_{{\text{0}}}$ is the laser fluence and ${\text{q}}({\text{t}})$ is the power density history of the laser pulse^46^. Note that other physical and chemical processes, such as photodesorption, annealing, and soot oxidation, may arise during particle cooling, but can be considered negligible when low or moderate laser fluences are used^21,47^, which is the case of the present work. The different processes influencing the soot particles temperature decay rates given by Eq. (1) should be adequately modeled in order to allow particle diameters to be determined from the measured temperature history. In the Rayleigh limit, the absorption cross section is given by:

2 $${\text{C}}_{{\text{a}}} = \frac{{\pi ^{2} {\text{d}}_{{\text{s}}}^{3} {\text{E}}({\text{m}})}}{\lambda },$$

where ${\text{E}}({\text{m}})$ is the soot absorption function^48,49^, and $\lambda$ represents the laser irradiation wavelength. The energy lost by thermal radiation is given by^47^:

3 $${{\dot{\text{q}}}}_{{\text{r}}} = 8\pi ^{3} {\text{d}}_{{\text{s}}}^{3} {\text{E}}({\text{m}})\frac{{{\text{k}}^{5} }}{{{\text{h}}^{4} {\text{c}}^{3} }}{\text{T}}^{5} {\text{N}}_{{\text{p}}} \int_{0}^{\infty } {\frac{{{\text{t}}^{4} }}{{e^{{\text{t}}} - 1}}} {\text{dt}},$$

where h, k and c are the Planck and the Boltzmann constants and the speed of light, respectively. ${\text{N}}_{{\text{p}}}$ is the aggregate size^46,50^ and the integration yields a constant value of 24.886^51^. Under ambient pressure conditions, the energy lost by thermal radiation can be considered negligible^22,51^. Using the hypothesis of free-molecular regime for the soot particle cooling by conduction^50^ and the Fuchs approach^52^, the conduction cooling rate of soot particles may estimated as:

4 $${{\dot{\text{q}}}}_{{\text{c}}} = \alpha \pi \left( {\frac{{{\text{d}}_{{\text{s}}} }}{2}} \right)^{2} \frac{{{\text{p}}_{{\text{g}}} }}{2}\sqrt {\frac{{8{\text{kT}}_{{\text{g}}} }}{{\pi {\text{m}}_{{\text{g}}} }}} \left( {\frac{{\gamma ^{*} + 1}}{{\gamma ^{*} - 1}}} \right)\left( {\frac{{\text{T}}}{{{\text{T}}_{{\text{g}}} }} - 1} \right),$$

where $\alpha$, ${\text{p}}_{{\text{g}}}$ and ${\text{T}}_{{\text{g}}}$ are, respectively, the soot thermal accommodation coefficient assumed as 0.37^46^, the ambient gas pressure and the temperature^6^. The mass of the surrounding gas molecule is ${\text{m}}_{{\text{g}}}$ and $\gamma ^*$ represents the average value of the surrounding gas specific heats ratio^50^. The energy lost due to particle sublimation may be modeled as^22,29^:

5 $${{\dot{\text{q}}}}_{{\text{s}}} = - \frac{{\Delta {\text{H}}_{{\text{v}}} }}{{{\text{M}}_{{\text{v}}} }}\frac{{{\text{dM}}}}{{{\text{dt}}}},$$

where ${\text{M}}_{{\text{v}}}$ is the soot molecular weight and $\Delta {\text{H}}_{{\text{v}}}$ represents the enthalpy of formation of carbon clusters. The particle mass loss rate by sublimation, ${\text{dM}}/{\text{dt}}$, may be written as^22,29^:

6 $$\frac{{{\text{dM}}}}{{{\text{dt}}}} = - \frac{{\pi {\text{d}}_{{\text{s}}} {\text{W}}_{{\text{v}}} \alpha _{{\text{M}}} {\text{p}}_{{\text{v}}} }}{{{\text{R}}_{{\text{p}}} {\text{T}}}}\left( {\frac{{{\text{R}}_{{\text{m}}} {\text{T}}}}{{2\pi {\text{W}}_{{\text{v}}} }}} \right)^{{1/2}} ,$$

where $\alpha _{{\text{M}}}$ is the mass accommodation coefficient, ${\text{p}}_{{\text{v}}}$ is the average partial pressure, ${\text{R}}_{{\text{p}}}$ and ${\text{R}}_{{\text{m}}}$ are the universal gas constant expressed in different units and ${\text{W}}_{{\text{v}}}$ is the average mass of the sublimed clusters^29^. Following^53,54^, the soot primary particles diameter probability distribution within the probe volume can be assumed as exhibiting a *lognormal* size distribution. i.e.,

7 $${\text{p}}({\text{d}}_{{\text{s}}} ) = \frac{1}{{{\text{d}}_{{\text{s}}} \sqrt {2\pi } {\text{ln}}~\sigma _{{\text{g}}} }}{\text{exp}}\left[ { - \left( {\frac{{{\text{ln}}~({\text{d}}_{{\text{s}}} /{\text{d}}_{{{\text{pg}}}} )}}{{\sqrt 2 {\text{ln}}~\sigma _{{\text{g}}} }}} \right)}^2 \right],$$

where ${\text{d}}_{{{\text{pg}}}}$ and $\sigma _{{\text{g}}}$, represent the geometric mean particle diameter and the geometric standard deviation, respectively. The primary particle diameter, ${\text{d}}_{{\text{s}}}$, has been obtained from the first moment of the *lognormal* distribution for given ${\text{d}}_{{{\text{pg}}}}$ and $\sigma _{{\text{g}}}$ values, following

8 $${\mathbb{E}}[{\text{d}}_{{\text{s}}}^{1} ] = {\text{exp}}\left[ {{\text{ln}}({\text{d}}_{{{\text{pg}}}} ) + \frac{{[{\text{ln}}(\sigma _{{\text{g}}} )]^{2} }}{2}} \right].$$

If the laser probe volume is small enough to allow for the assumption of an optically thin path, and the soot particles are uniformly distributed, the modeled total thermal emission intensity of this particle distribution at wavelength $\lambda _i$ may be expressed as^28^:

9 $${\text{TEI}}_{{\text{i}}} \propto \int_{0}^{\infty } p ({\text{d}}_{{\text{s}}} )\frac{{2\pi {\text{c}}^{2} {\text{h}}}}{{\lambda _{{\text{i}}}^{5} }}\left[ {\exp \left( {\frac{{{\text{hc}}}}{{\lambda _{{\text{i}}} {\text{kT}}({\text{d}}_{{\text{s}}} )}}} \right) - 1} \right]^{{ - 1}} \frac{{\pi ^{2} {\text{d}}_{{\text{s}}}^{3} {\text{E}}({\text{m}}_{{\text{i}}} )}}{{\lambda _{{\text{i}}} }}~{\text{d}}({\text{d}}_{{\text{s}}} ),$$

where ${\text{T}}({\text{d}}_{{\text{s}}} )$ is the solution of Eq. (1). Then, the theoretical effective temperature time-history may be obtained from the ratio of total thermal emission at two different wavelengths ($\lambda _1>\lambda _2$), by using the two-color pyrometry equation^38^:

10 $${\text{T}}_{{\text{e}}} ({\text{d}}_{{{\text{pg}}}} ,\sigma _{{\text{g}}} ) = \frac{{{\text{hc}}}}{{\text{k}}}{\text{C}}_{2} \left( {\frac{1}{{\lambda _{2} }} - \frac{1}{{\lambda _{1} }}} \right)\left/\mathord{\vphantom{\frac{{\int_{0}^{\infty } p ({\text{d}}_{{\text{s}}} ){\text{d}}_{{\text{s}}}^{3} \exp [ - {\text{C}}_{2} /\lambda _{2} {\text{T}}({\text{d}}_{{\text{s}}} )]{\text{d}}({\text{d}}_{{\text{s}}} )}}{{\int_{0}^{\infty } p ({\text{d}}_{{\text{s}}} ){\text{d}}_{{\text{s}}}^{3} \exp [ - {\text{C}}_{2} /\lambda _{1} {\text{T}}({\text{d}}_{{\text{s}}} )]{\text{d}}({\text{d}}_{{\text{s}}} )}}}}\right.\ln \frac{{\int_{0}^{\infty } p ({\text{d}}_{{\text{s}}} ){\text{d}}_{{\text{s}}}^{3} \exp [ - {\text{C}}_{2} /\lambda _{2} {\text{T}}({\text{d}}_{{\text{s}}} )]{\text{d}}({\text{d}}_{{\text{s}}} )}}{{\int_{0}^{\infty } p ({\text{d}}_{{\text{s}}} ){\text{d}}_{{\text{s}}}^{3} \exp [ - {\text{C}}_{2} /\lambda _{1} {\text{T}}({\text{d}}_{{\text{s}}} )]{\text{d}}({\text{d}}_{{\text{s}}} )}},$$

where ${\text{C}}_{2} = {\text{hc/k}}$ is the second Planck constant and the Wien approximation, exp(${\text{hc/k}}\lambda {\text{T}}$)$\gg$ 1, has been employed. On the other hand, after the laser pulse, the effective temperature time history of the soot particle distribution can be determined from the measured TiRe-LII signal as:

11 $${\text{T}}_{{{\text{e}},{\text{exp}}}} = \frac{{{\text{hc}}}}{{\text{k}}}\left( {\frac{1}{{\lambda _{2} }} - \frac{1}{{\lambda _{1} }}} \right)\left[ {\ln \left( {\frac{{{\text{S}}_{{{\text{LII}},\lambda _{1} }} {\text{E}}({\text{m}}_{2} )\lambda _{1}^{6} }}{{{\text{S}}_{{{\text{LII}},\lambda _{2} }} {\text{E}}({\text{m}}_{1} )\lambda _{2}^{6} }}} \right)} \right]^{{ - 1}} ,$$

where the two-color pyrometry relation has also been used. Furthermore, the Sauter mean diameter (${\text{d}}_{{32}}$) may be related to the initial temperature decay rate as follows^28^:

12 $$\left.\frac{{{\text{dT}}_{{\text{e}}} }}{{{\text{dt}}}}\right|_{{{\text{t = }}\tau _{{{\text{max}}}} }} = - \frac{{\Theta ({\text{T}}_{{{\text{max}}}} - {\text{T}}_{{\text{0}}} )}}{{{\text{d}}_{{32}} }},$$

which is evaluated at time (${\text{t = }}\tau _{{{\text{max}}}}$) where the effective temperature peak (${\text{T}}_{{{\text{max}}}}$) is observed, which typically occurs 20 ns after the laser pulse^28,32,33^. Also, ${\text{T}}_{{\text{0}}}$ represents the initial soot temperature, which may be determined by using a two color pyrometry technique, in the absence of laser irradiation. The parameter $\Theta$ represents a cluster of all the surrounding gas and soot particles thermal properties^28^. Under the assumption of a *lognormal* soot particle diameter probability distribution, the Sauter mean diameter (${\text{d}}_{{32}}$) is related to the two distribution parameters (${\text{d}}_{{{\text{pg}}}}$, $\sigma _{{\text{g}}}$) by:

13 $${\text{d}}_{{32}} = {\text{d}}_{{{\text{pg}}}} \exp \left[ {2.5(\ln \sigma _{{\text{g}}} )^{2} } \right].$$

These soot particle distribution parameters ($${\text{d}}_{{{\text{pg}}}}$$, $$\sigma
_{{\text{g}}}$$) are estimated by minimizing the difference between the computed and measured values of the soot effective temperature, $${\text{T}}_{{\text{e}}}$$, according to the iterative process shown in the Fig.  8b. Indeed, the Sauter mean diameter ($${\text{d}}_{{32}}$$) obtained from the experimental effective temperature (step 2), Eq. (12), is used as an input parameter to compute a modeled effective soot temperature, Eq. (10), and its corresponding *lognormal* distribution parameters (step 3), $${\text{d}}_{{{\text{pg}}}}$$ and $$\sigma _{{\text{g}}}$$, which are related via Eq. (13). For this purpose, Eq. (1) is numerically solved within a range of spherical soot primary particle diameters (1 to 105 nm)^28^  (step 1). The difference between the numerical and experimental effective temperature decay is then iteratively reduced until reaching a minimum error. The quality of the parameters estimation results is evaluated by considering the absolute error ($$\epsilon$$), which compares the predicted and measured effective temperature values at each the flame location:

14 $$\epsilon = \left| {{\text{T}}_{{{\text{e}},{\text{exp}}}} - {\text{T}}_{{\text{e}}} ({\text{d}}_{{{\text{pg}}}} ,\sigma _{{\text{g}}} )} \right|.$$

In addition, the Chi-Squared test ($\chi ^2$) also is used as a suitable test for the purpose of evaluating the effective temperatures agreement^53^:

15 $$\chi ^{2} = \sum\limits_{{i = 1}}^{N} {\frac{{\left[ {{\text{T}}_{{{\text{e}},{\text{exp}},{\text{i}}}} - {\text{T}}_{{{\text{e}},i}} ({\text{d}}_{{{\text{pg}}}} ,\sigma _{{\text{g}}} )} \right]^{2} }}{{\sigma _{{\text{g}}}^{2} }}}.$$

where *N* represents the number of experimental TiRe-LII measurements used to determine the effective temperature history.

### Experimental apparatus

**Fig. 9:**
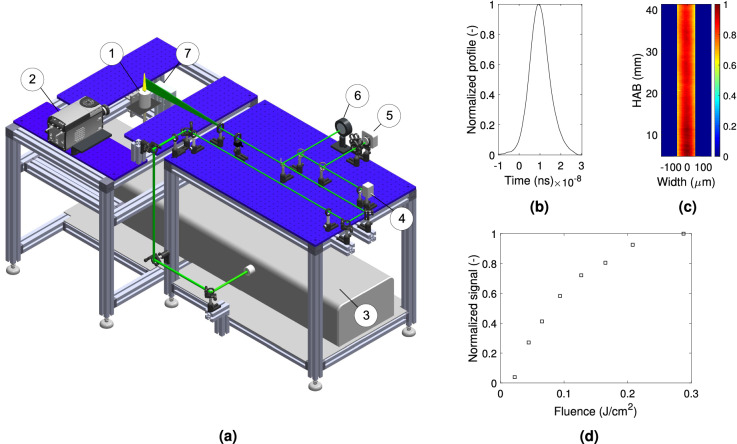
(**a**) Schematic of the experimental set-up for the TiRe-LII diagnostic and the main devices: (1) Candle; (2) ICCD camera; (3) Nd:YAG laser; (4) Fast photodiode; (5) Beam profiler, (6) Laser energy sensor and (7) Linear stage. Beam characterization: (**b**) Laser power temporal profile at a fluence of $0.148\;{\text{J/cm}}^{2}$. (**c**) Laser sheet thickness and (**d**) mean normalized signal for different fluences at HAB $\approx 22\;{\text{m}}$. The visible height of the flame is ${\text{L}}_{{\text{f}}} = 38\;{\text{mm}}$.

The experimental setup used for the TiRe-LII measurements is shown in Fig. 9a. In the adopted configuration, the second harmonic (532 nm) of a Nd:YAG Litron Aurora II laser (3), with a 9 mm beam diameter and 10 Hz repetition rate, has been used to excite the incandescence (${\text{S}}_{{{\text{LII}}}}$) of the soot particles formed in the candle flame (1). The candles are composed of Sasolwax 6203 paraffin, and have been manufactured in house according to procedures detailed elesewhere^6^. These candles are characterized by wick diameter and length values of ${\text{D}}_{{{\text{wick}}}}$ = 3 mm and ${\text{L}}_{{{\text{wick}}}}$ = 7 mm, respectively. These wick dimensions have been chosen since they correspond to the stablest experiments previously performed^6^, and lead to flames that operate below the smoke point. The height of the candle flame measured from the wax pool (${\text{L}}_{{\text{f}}}$) is consistent with previous measurements^4,6^, which have been performed for ${\text{D}}_{{{\text{wick}}}} = 3\;{\text{mm}}$ and ${\text{L}}_{{{\text{wick}}}} = 7\;{\text{mm}}$.

As evidenced by Eq. (1), the accurate characterization of the laser pulse is paramount for the TiRe-LII technique. Accordingly, Fig. 9b shows the temporal profile of a typical laser pulse (8 ns span), which has been measured with a fast photodiode ET-2030 (4) coupled to a 1 GHz oscilloscope (LeCroy Wavesurfer 3104Z). An iris is employed to select a 6 mm diameter central portion of the laser beam that is expanded, with the help of a spherical convex lens ($f = 750\,\hbox {mm}$) and a concave cylindrical lens ($f = - 50\,\hbox {mm}$) to form a thin laser sheet, which crosses the flame centerline. As observed in Fig. 9c, the sheet thickness is $120~\upmu\hbox {m}$, whereas its height is 65 mm. Note that the sheet thickness is roughly $1/10^{{{\text{th}}}}$ of the maximum flame diameter, 2 mm. To correct both for the laser intensity non-homogeneity and for the shot-to-shot laser fluctuations, the corresponding spatial and temporal energy distributions are respectively mapped with a beam profiler (Coherent LaserCam-HR II) (5) and by an energy sensor (Coherent J-50MB-YAG) (6), which is coupled to a Coherent Labmax TOP energy meter.

The laser incandescence signal emitted by the soot particles is captured with an intensified CCD camera (Andor Istar DH334T) with a $1024 \times 1024$ px$^2$ matrix (2), that is coupled with a Nikon AF Nikkor 50 mm lens (*f*/1.4). The LII signal first passes through narrow band filters (40 nm FWHM) centered either at 450 nm or at 650 nm, which have been selected in order to improve the signal to noise ratio. All the measurement devices are synchronized with an external (Quantum sapphire 9200) pulse generator (not show here). A motorized linear stage (7) has been used to progressively raise the candle as the wax is consumed, thus ensuring that the flame lies at the same measurement region during all tests. Since the TiRe-LII technique requires for the laser illuminated soot particles to be heated below the carbon sublimation temperature, care must be taken when selecting the laser fluence. Accordingly, the normalized fluence curve of the candle flame, shown in Fig. 9d, indicates that the plateau region should be achieved beyond $0.16\;{\text{J/cm}}^{2}$. Then, in order to reduce the soot sublimation effect, a laser sheet fluence of $0.148\;{\text{J/cm}}^{2}$ has been used in this work to obtain the ${\text{S}}_{{{\text{LII}}}}$. In order to improve the signal-to-noise-ratio, $2 \times 2$ pixel binning has been used, thus resulting in an image resolution of 12.6 px/mm. For each measurement delay after the laser pulse, 200 images have been captured at each wavelength. The effective soot temperature uncertainty may be estimated following previous works^55^, and is attributed to the wavelength separation^38^, the standard deviation of incandescence images due to inherent noise of the ICCD cameras, and the value of absorption function, which is currently matter for debate^21,49^. It should also be stressed that, to the best of the authors knowledge, the value of ${\text{E}}({\text{m}})$ in candle flames has not been previously reported. Therefore, a classical correlation^48^ is assumed to hold in this study.
